# The Potential Mechanisms of Arrhythmia in Coronavirus disease-2019

**DOI:** 10.7150/ijms.94578

**Published:** 2024-05-19

**Authors:** Jianhong Li, Qiuyuan Huang, Yifan Liang, Jun Jiang, Yan Yang, Jian Feng, Xiaoqiu Tan, Tao Li

**Affiliations:** 1 Key Laboratory of Medical Electrophysiology of the Ministry of Education, Medical Electrophysiological Key Laboratory of Sichuan Province, Institute of Cardiovascular Research, Southwest Medical University, Luzhou 646000, China.; 2 Department of Cardiology, The Affiliated Hospital of Southwest Medical University, Luzhou 646000, China.; 3 Department of Physiology, School of Basic Medical Sciences, Southwest Medical University, Luzhou 646000, China.; 4 Department of General Surgery (Thyroid Surgery), The Affiliated Hospital of Southwest Medical University, Luzhou 646000, China.

**Keywords:** SARS-CoV-2, COVID-19, Arrhythmia, Cytokine storm

## Abstract

Severe acute respiratory syndrome coronavirus-2 (SARS-CoV-2) leads to coronavirus disease-2019 (COVID-19) which can cause severe cardiovascular complications including myocardial injury, arrhythmias, acute coronary syndrome and others. Among these complications, arrhythmias are considered serious and life-threatening. Although arrhythmias have been associated with factors such as direct virus invasion leading to myocardial injury, myocarditis, immune response disorder, cytokine storms, myocardial ischemia/hypoxia, electrolyte abnormalities, intravascular volume imbalances, drug interactions, side effects of COVID-19 vaccines and autonomic nervous system dysfunction, the exact mechanisms of arrhythmic complications in patients with COVID-19 are complex and not well understood. In the present review, the literature was extensively searched to investigate the potential mechanisms of arrhythmias in patients with COVID-19. The aim of the current review is to provide clinicians with a comprehensive foundation for the prevention and treatment of arrhythmias associated with long COVID-19.

## Introduction

The novel coronavirus disease-2019 (COVID-19) is a severe acute respiratory syndrome caused by coronavirus-2, also known as severe acute respiratory syndrome coronavirus-2 (SARS-CoV-2) 1, 2. Since the first case was reported in Wuhan, China, in December 2019, it has rapidly escalated into a global pandemic 3, inflicting significant harm on people around the world and placing an enormous burden on healthcare workers 4. Thanks to the concerted efforts of people around the world, remarkable results have been achieved in the prevention and control of the novel coronavirus pneumonia epidemic 5. The widespread vaccination with the novel coronavirus vaccine and the weakening of the virulence of the novel coronavirus 6, 7, led to a more stable situation 5, 8. However, it is important to recognize that a risk of increased infection rates remains 8, 9. The individuals who are particularly susceptible to the virus include those with underlying health issues such as cardiovascular diseases, diabetes, as well as the elderly population 10, 11.

The primary system affected by COVID-19 is the respiratory system, with respiratory symptoms being prevalent 12. The severity of the disease can range from asymptomatic or mild upper respiratory tract infections to life-threatening conditions such as acute respiratory distress syndrome (ARDS) and respiratory failure 2, 13. It is noteworthy that respiratory failure has emerged as a leading cause of mortality associated with COVID-19 13. With the emergence of a large number of clinical cases, it has been shown that the cardiovascular system is also vulnerable 14. Numerous patients experience complications related to serious heart diseases, such as myocardial injury, arrhythmias, acute coronary syndrome and others 11, 14, 15. Previous studies have indicated that 20-40% of hospitalized cases exhibit evidence of myocardial injury, such as cardiogenic chest pain, fulminant heart failure, arrhythmias and cardiogenic death 16.

Studies have demonstrated that COVID-19 can lead to myocardial damage and arrhythmia, exacerbating the condition of patients with pre-existing cardiovascular diseases, and resulting in poor prognosis and increased mortality rates 11, 12, 17. Among them, arrhythmia is a serious and life-threatening condition following SARS-CoV-2 infection 10. A survey of hospitalized patients with COVID-19 revealed that 19.6% of patients with COVID-19 experienced arrhythmias, which was a major complication 18. The prevalence of arrhythmias in patients admitted to intensive care units was even higher reaching 44.4% 18. According to a clinical bulletin issued by the American College of Cardiology, the incidence of cardiac arrhythmias in hospitalized patients with COVID-19 is about 16.7% 19. The exact mechanism of arrhythmic complications in patients with COVID-19 is complex and not well understood 20, 21. Thus, in the present comprehensive review, the literature was extensively searched to investigate the mechanisms underlying cardiac arrhythmias associated with COVID-19. The aim of the current review is to provide clinicians with valuable insights for optimizing treatment plans, management strategies, including those for post-acute COVID-19 syndrome, as well as exploring potential targets and diagnostic markers associated with cardiac arrhythmias in the context of COVID-19.

## SARS-CoV-2 and angiotensin-converting enzyme 2 (ACE2)

**Figure 1:**
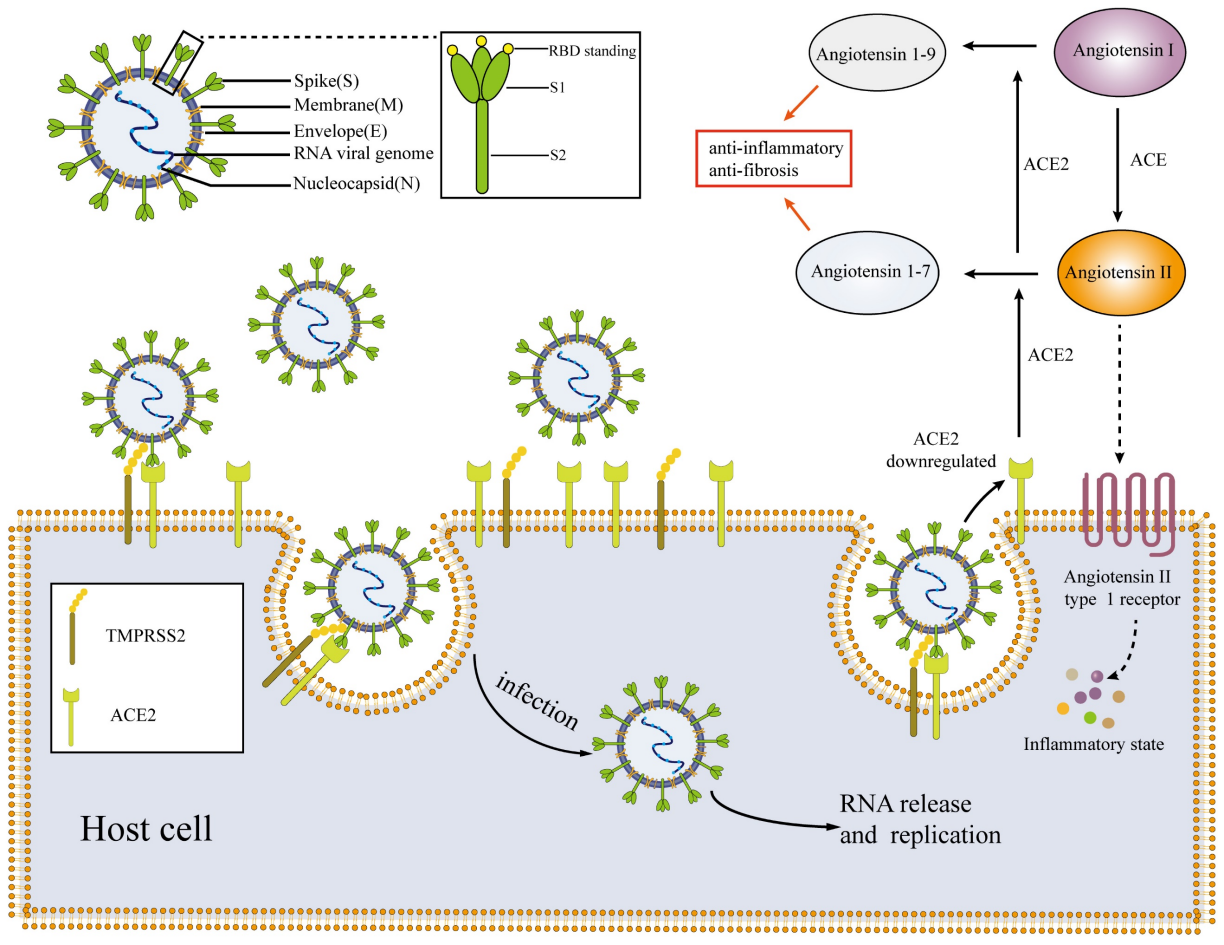
Mechanisms and pathophysiology of SARS-CoV-2. SARS-CoV-2 enters host cells through the interaction of the S1 subunit with ACE2 and the interaction of S2 with TMPRSS2; the virus then replicates and proliferates within host cells. After SARS-CoV-2 invades the body, the expression of ACE2 is downregulated, resulting in an imbalance of the RASS system and an inflammatory environment. SARS-CoV-2: severe acute respiratory syndrome coronavirus-2; ACE: angiotensin-converting enzyme; TMPRSS2: trans membrane protease serine 2; RBD: receptor binding domain.

SARS-CoV-2 falls within the β coronavirus group and belongs to the larger coronavirus subfamily 22. This positive sense single-stranded RNA virus consists of particles enclosed in an envelope 16. The morphology of the virus particles is typically round or elliptical, with a diameter range of 60-140 nm 23. The primary components of these particles are positive sense single-stranded RNA and structural proteins 23. Recent studies have revealed the presence of four essential structural proteins within the virus particles, including the spike protein S, envelope protein E, membrane protein M and nucleocapsid protein N (**Figure 1**) 22, 24. The nucleocapsid protein N encapsulates the viral RNA forming the core part of the viral particle, known as the nucleocapsid. The nucleocapsid is enclosed within a lipid membrane consisting of two layers. The S, M and E proteins of the novel coronavirus are embedded within the lipid membrane 16, 23, 24. Among these proteins, the S protein plays a crucial role in facilitating virus entry into host cells. It belongs to the class I fusion glycoprotein family and exists as a homotrimer. The S protein has two functional subunits, named S1 and S2 22. The S1 subunit comprises the N-terminal domain and the receptor binding domain (RBD) 25. The primary function of the S1 subunit is binding site of receptors on host cells 22, 26. In the case of the novel coronavirus, the RBD on the S1 subunit recognizes and interacts with the ACE2 presented on host cells 27, 28. The S2 subunit facilitates the fusion of the viral and host membranes following the binding of the S1 subunit to the ACE2, primarily through its interaction with the trans membrane protease serine 2 (TMPRSS2) 23, 24, 29. To facilitate viral replication and proliferation within the host, the presence of ACE2 and TMPRSS2 co-expression on the surface of host cells is a prerequisite for SARS-CoV-2 to invade and infect these cells 12. Therefore, ACE2, TMPRSS2, S1 and S2 may be potential targets for the treatment of COVID-19 22, 30.

It is noteworthy that the novel coronavirus has the ability to infect various mammalian species, including humans 3, 31. Bats likely serve as the natural reservoir of SARS-CoV-2, while pangolins may act as intermediate hosts 32. Additionally, golden hamsters have been found to be susceptible to SARS-CoV-2 infection, whereas there is no evidence suggesting that the virus can infect dogs, chickens, ducks, or pigs 3, 16. High temperatures (> 56 ºC), ultraviolet irradiation, 75% ethanol, 60% isopropanol, diethyl ether, peracetic acid and chlorine-containing disinfectants can effectively inactivate SARS-CoV-2 33. In both clinical settings and daily life, 75% ethanol and chlorine-containing disinfectants are commonly used to deactivate the novel coronavirus 33. However, it has been observed that chlorhexidine does not effectively inactivate the virus 33.

ACE2 serves as a crucial target for SARS-CoV-2 to invade host cells, and is involved in various pathophysiological processes of COVID-19. It plays a key role in the pathogenesis of SARS-CoV-2 34, 35. ACE2 is an important component of the renin-angiotensin-aldosterone system (RAAS) and acts as a protective factor in the body 16, 36. ACE2 is mainly found in type II lung cells, macrophages, perivascular cells and cardiomyocytes 14, 16, 31. In RAAS, ACE2 primarily converts angiotensin I to angiotensin 1-9 and angiotensin II to angiotensin 1-7 16, 36. Angiotensin 1-9 and angiotensin 1-7 have a protective effect on the body, promoting vasodilation, exhibiting anti-inflammatory and anti-fibrotic properties which can counteract the inflammatory effects of RAAS 12, 16. By contrast, angiotensin II activates inflammatory responses through angiotensin type 1 (AT1) receptors 21. When SARS-CoV-2 infects host cells, it binds to ACE2 presented on the host cell membrane (**Figure 1**) 29. Viruses compete for binding and inhibit ACE2 binding to cells 29, resulting in decreased production of angiotensin 1-9 and angiotensin 1-7, while the levels of angiotensin II accumulate 21, 29. Such alterations can have detrimental effects on the body, particularly on the cardiovascular system 12, 13, 21. The angiotensin converting enzyme inhibitors (ACEIs) and the angiotensin receptor blockers (ARBs) are commonly prescribed for the management of cardiovascular diseases 37, 38. They serve as secondary prevention medications following conditions such as coronary atherosclerotic heart disease, chronic heart failure, and myocardial infarction 37. These drugs block angiotensin II, thereby promoting vasodilation, reducing blood pressure, thus preventing further heart damage 38, 39. RAAS inhibitors primarily inhibit the RAAS axis, which can potentially result in the upregulation of the expression level of ACE2 and increased ACE2 activity. This, in turn, may increase the susceptibility of patients to COVID-19, posing potential harm to the body 16. However, there is currently no evidence to support the notion that ACEIs or ARBs can upregulate ACE2 levels in human tissues or markedly impact the prognosis of patients with COVID-19 40. By contrast, some studies suggest that ACEIs or ARBs administration may even have a protective effect by preventing the virus from infecting cells. Consequently, the effects of RAAS inhibitors on patients with COVID-19 remain controversial, resembling a 'double-edged sword' scenario 40, 41.

With an in-depth study of the infection mechanism of COVID-19, Baggen *et al.* 42 found that COVID-19 can also infect cells through other ways that do not depend on the ACE2. In that study, it was revealed that a lysosomal protein called TMEM106B may be a new receptor for SARS-CoV-2. This protein can mediate viral entry in cells with low or no ACE2 expression cells such as brain cells, immune cells and lung adenocarcinoma cells, becoming a substitute receptor for ACE2 42. This discovery may provide a new approach to the treatment of COVID-19. Understanding the pathophysiology of SARS-CoV-2 can enhance our knowledge of the pathogenic mechanism of the virus and the potential mechanisms underlying COVID-19-associated arrhythmia 43, thereby aiding researchers and clinicians in identifying novel targets for intervention and strategies for preventing and treating arrhythmias related to COVID-19.

## Potential Mechanisms of Arrhythmias in Patients with COVID-19

Arrhythmia is a frequent cardiovascular complication in patients with COVID-19, posing a potential threat to their lives 10, 14. Arrhythmias observed in patients with COVID-19 include various types such as tachyarrhythmias and bradyarrhythmias. Tachyarrhythmias can be further divided into supraventricular and ventricular tachycardia. Supraventricular tachycardia includes sinus tachycardia, atrial premature beats, atrial flutter, atrial fibrillation and paroxysmal supraventricular tachycardia. Ventricular tachycardia includes ventricular premature beats, non-sustained ventricular tachycardia, sustained ventricular tachycardia, polymorphic ventricular tachycardia or torsade de pointes (Tdp) and ventricular fibrillation. Bradyarrhythmias include sinus bradycardia and atrioventricular block, which can be divided into first, second and third degree atrioventricular block (complete heart block), bundle branch block, no pulse electrical activity and cardiac arrest 21, 44, 45. According to studies conducted by the Heart Rhythm Society, atrial fibrillation is the most common tachyarrhythmia observed in patients with COVID-19 (21%), while severe sinus bradycardia (8%) and complete heart block (8%) are the most frequent chronic arrhythmias observed in hospitalized patients with COVID-19 45. Atrial flutter and paroxysmal supraventricular tachycardia accounted for 5.4 and 5.7% of COVID-19-related arrhythmias, respectively. Frequent monomorphic premature ventricular contractions and polymorphic premature ventricular contracts represented 5.3 and 3.5% of COVID-19-related arrythmias, respectively. Non-sustained ventricular tachycardia and sustained ventricular tachycardia represented 6.3% and 3.8% of COVID-19-related arrythmias, respectively. Polymorphic ventricular tachycardia or TdP, first or second-degree AV block, bundle branch block, pulseless electrical activity and ventricular fibrillation or cardiac arrest represented 3.5, 5.9, 3.9, 5.6 and 4.8% of COVID-19-related arrythmias, respectively 45.

**Figure 2:**
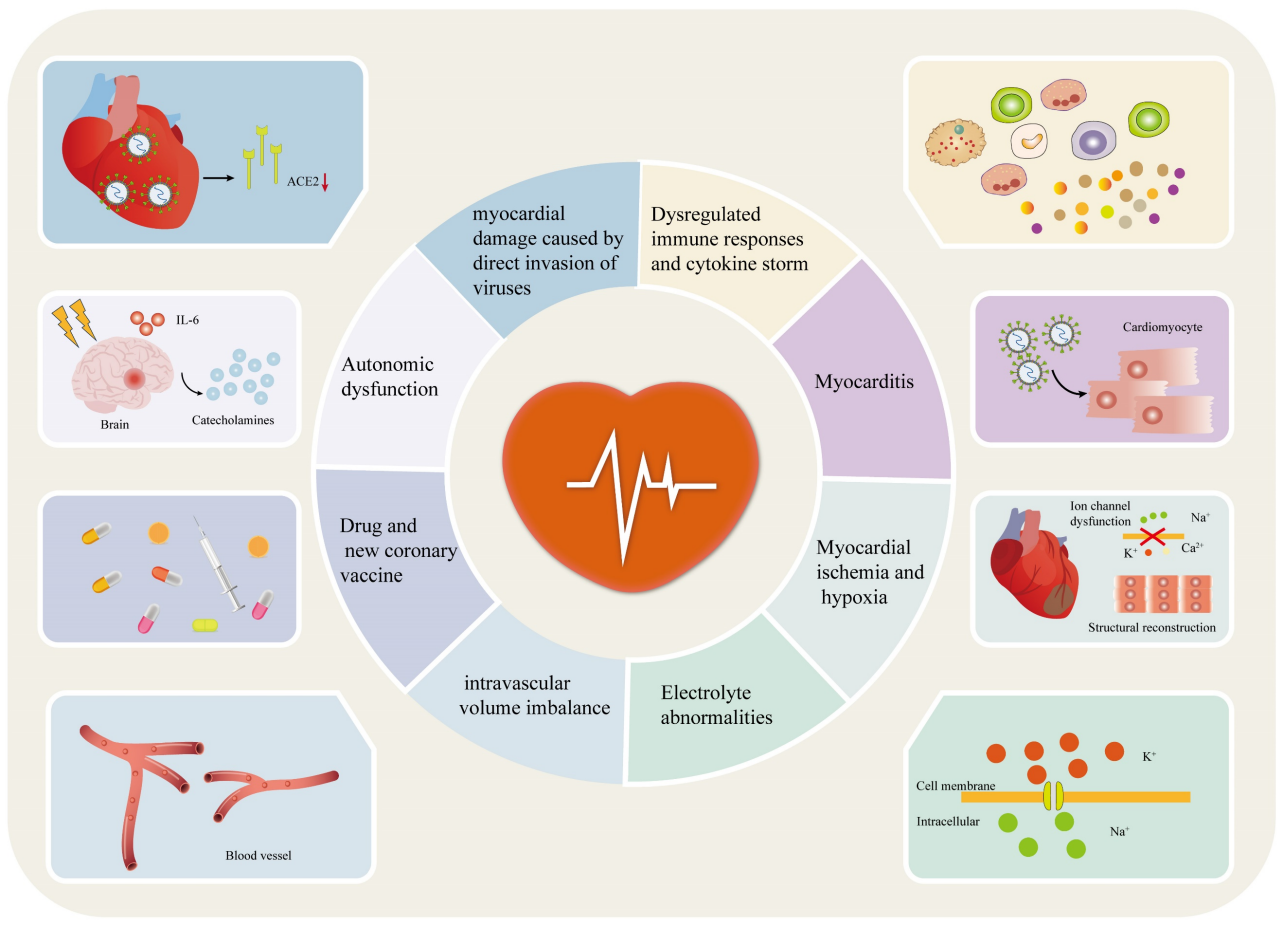
Potential mechanisms of arrhythmia in patients with COVID-19. Potential mechanisms of arrhythmia in patients with COVID-19 include myocardial damage caused by direct invasion of viruses, myocarditis, immune response dysregulation and cytokine storm, myocardial ischemia/hypoxia, electrolyte abnormalities, intravascular volume imbalances, drug interactions, COVID-19 vaccines and autonomic nervous dysfunction. ACE2: angiotensin-converting enzyme 2; IL-6: interleukin 6; COVID-19: coronavirus disease-2019.

At present, the exact mechanism behind the occurrence of arrhythmia in patients with COVID-19 is not fully understood 20, 21. However, numerous studies suggest that it may be associated with factors such as myocardial damage caused by direct invasion of viruses, myocarditis, immune response dysregulation and cytokine storm, myocardial ischemia/hypoxia, electrolyte abnormalities, intravascular volume imbalances, drug interactions, COVID-19 vaccines, and autonomic nervous dysfunction (**Figure 2**) 10, 12, 23, 46-48.

### Myocardial damage caused by direct invasion of viruses

Because ACE2 is expressed at high levels in lung and heart tissue, SARS-CoV-2 can directly infect and damage myocardial tissue as well as lung tissue 49. Through autopsy case analysis, the presence of the novel coronavirus was observed in the cardiac tissue of patients with COVID-19. Similarly, electron microscopy of endocardial tissue biopsy confirmed the presence of SARS-CoV-2 particles in myocardial tissue 50. Han *et al.* 49 also found SARS-CoV-2 presented in hamster sinoatrial node cells. All of results showed that SARS-CoV-2 may directly invade and infect myocardial tissue. The myocardial injury caused by direct invasion of the virus is mainly due to the direct toxicity of the virus and the imbalance of ACE2 expression 13, 16.

The virus enters the cell by binding to ACE2, leading to imbalance of ACE2 expression, accumulation of angiotensin II, and decrease of angiotensin 1-7 and 1-9, resulting in RASS system disorders and causing myocardial injury 50. At the same time, after the virus enters myocardial cells, auxiliary proteins such as spike proteins can cause cell degeneration and necrosis, leading to myocardial damage 50. Evidence shows that the prevalence of arrhythmia in COVID-19 patients with myocardial injury is far higher than that without myocardial injury 10, 49. Myocardial injury caused by direct invasion of SARS-CoV-2 can lead to arrhythmia through multiple ways. Studies have shown that SARS-CoV-2 can directly damage cardiac conduction cells and cardiac working cells 40, 47. SARS-CoV-2 can invade cardiac conduction cells, such as sinoatrial node cells with pacing function, cause iron apoptosis in the cells and lead to sinoatrial node dysfunction, abnormal cardiac pacing rhythm and various arrhythmias 51. Myocardial injury can also cause Ca^2+^ influx in cardiac working cells, destroy the normal circulating Ca^2+^ influx peak, cause calcium homeostasis imbalance in myocardial cells, lead to calcium alternation and increase susceptibility to arrhythmia 44. At the same time, calcium homeostasis imbalance can also induce delayed depolarization, leading to arrhythmia 44. Inflammation caused by RASS system disorders and the toxic effects of the novel coronavirus can also cause cardiac tissue fibrosis and atrial enlargement, and myocardial structure reconstruction. It eventually leads to abnormal conduction of electrical activity of cardiomyocytes and promotes arrhythmia 13, 21, 44. The mechanism of arrhythmia caused by myocardial injury may be multifaceted and interactive requiring additional investigation 13.

### Dysregulated immune response and cytokine storm

**Figure 3:**
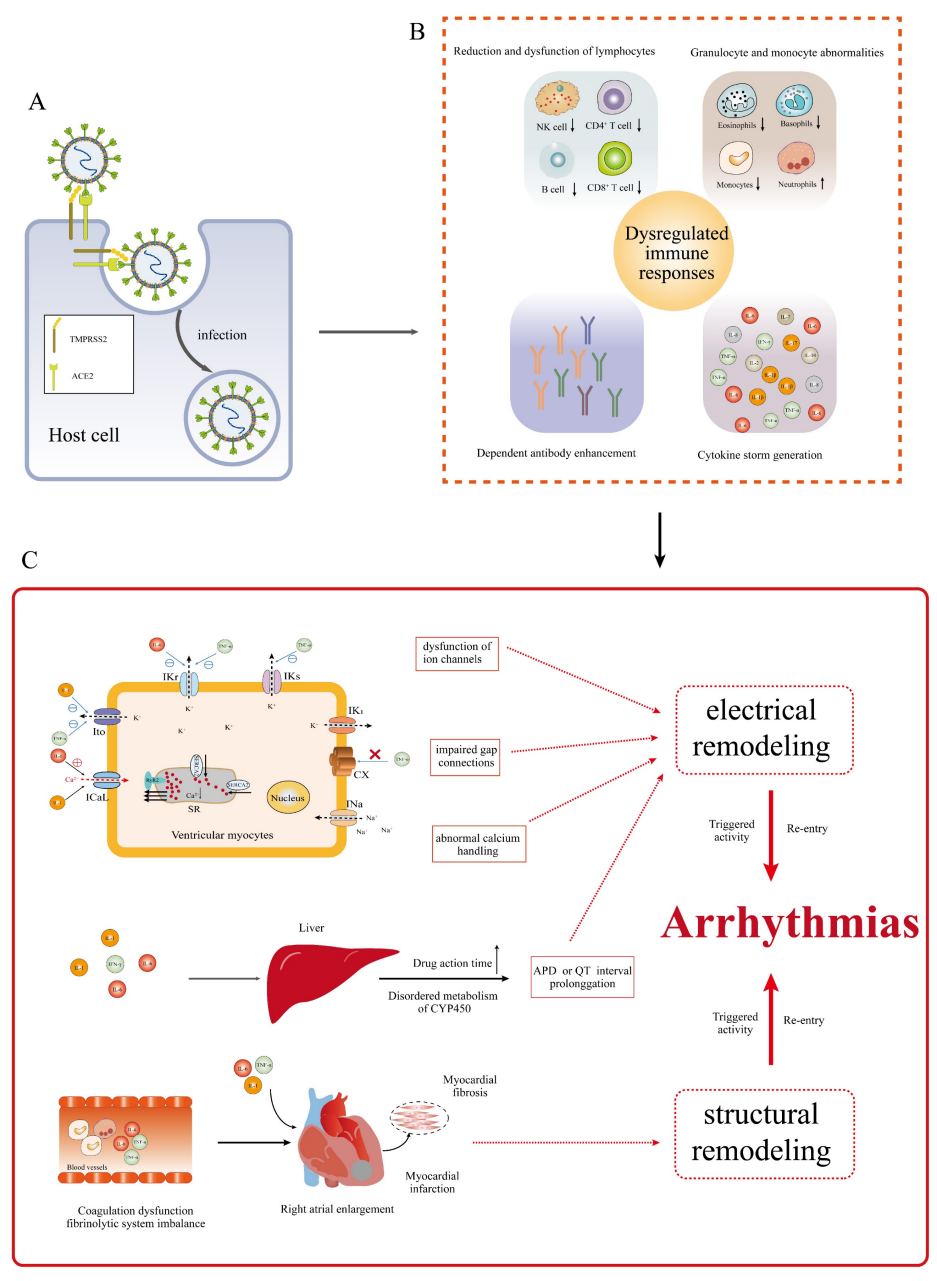
Mechanisms of abnormal immune response and cytokine storm leading to arrhythmia in patients with COVID-19. (A) SARS-CoV-2 invasion of host cells. (B) Mechanism of immune response dysregulation after SARS-CoV-2 invasion. The invasion of SARS-CoV-2 leads to lymphocytopenia, lymphocyte dysfunction, granulocyte and monocyte abnormalities, cytokine storm and dependent antibody enhancement. (C) Mechanisms of abnormal immune response and cytokine storm leading to arrhythmia in patients with COVID-19. The dysfunction of immune response and cytokine storm can lead to myocardial electrical remodeling and cardiac restructuring. Eventually, triggered activity and/or re-entry occur, promoting arrhythmias. IL: interleukin; IFN-γ: interferon-γ; TNF-α: tumor necrosis factor-α; IKr: rapid activation of delayed rectified potassium current; Ito: transient output potassium current; ICaL: L-Ca^2+^ channels; IKs: slow delayed rectifying potassium channel; CX: connexin; APD: action potential duration; RYR2: ryanodine receptor 2; SERCA: sarcoplasmic reticulum Ca^2+^-ATPase; COVID-19: coronavirus disease-2019; SARS-CoV-2: severe acute respiratory syndrome coronavirus-2; ACE2: angiotensin-converting enzyme 2; TMPRSS2: trans membrane protease serine 2; NK: natural killer cell; INa: Na^+^-current; SR: sarcoplasmic reticulum; CYP450: cytochrome P450.

The immune response caused by the invasion of the novel coronavirus is a notable link with the pathogenesis of pneumonia 16. After the novel coronavirus invades the body, it first activates the innate immune response, induces interferons, and the production of proinflammatory cytokines and chemokines, recruits immune cells to kill the virus and initiates adaptive immune response to clear the virus 23. If the virus is not cleared in time, the immune response is further activated leading to lymphocytopenia, mainly including CD4^+^ and CD8^+^ T lymphocytes, natural killer (NK) cells and B lymphocytes, dysfunction of T lymphocytes and NK cells, granulocyte and monocyte abnormalities including increase in neutrophils and decrease in eosinophils, basophils and monocytes, cytokine storm generation and dependent antibody enhancement; it eventually leads to immune system dysfunction (**Figure 3B**) 23, 48, 52. Among them, cytokine storm is the central link of immune dysregulation and the cause of multiple organ failure complicated by COVID-19 10. After the novel coronavirus infects the body, overactivated lymphocytes, macrophages, neutrophils and dendritic cells secrete a large number of cytokines such as interleukin (IL) 1, 2 , 6 , 7, 8, 10 and 17, granulocyte colony-stimulating factor, granulocyte macrophage colony-stimulating factor, interferon-induced protein-10, monocyte chemotactic protein 1, macrophage inflammatory protein-1α, interferon-γ (IFN-γ) and Tumor necrosis factor-α (TNF-α); it puts the body in a state of high inflammation, leading to cytokine storm 10, 23, 48, 52, 53.

Dysregulation of immune response and cytokine storm are marked causes of arrhythmia in patients with COVID-19 (**Figure 3C**) 23. The excessive production of inflammatory cytokines creates a state of high inflammation in the body, which can contribute to the occurrence of various arrhythmias, such as atrial fibrillation, long QT syndrome, TdP and atrioventricular block 54. Previous studies have also demonstrated the strong association between elevated levels of inflammatory cytokines, particularly IL-6 and IL-10, in the bloodstream of hospitalized patients with COVID-19 and the occurrence of atrial and ventricular arrhythmias 54, 55. Inflammatory cytokines have been shown to be involved in promoting the development of COVID-19 related arrhythmias through various mechanisms 54. Firstly, they can directly impact the function of ion channels in cardiomyocytes, resulting in their dysfunction and thereby promoting arrhythmias 54. This phenomenon is also referred to as inflammatory cardiac pathway diseases 55, 56. Monnerat *et al.* 10, 55, 57-60 demonstrated that inflammatory cytokines such as IL-1, IL-6 and TNF-α can regulate the function of ion channels such as K^+^ channel and Ca^2+^ channels on the myocardial membrane using the whole cell patch-clamp technique, resulting in the dysfunction of ion channels in cardiomyocytes, causing changes the duration of the action potential, leading to myocardial electrical remodeling and promoting arrhythmia. IL-6 can inhibit the rapid activation of delayed rectified potassium current (IKr) in cardiomyocytes, and IL-1 can inhibit its transient potassium outward current (Ito). IKr and Ito are the main ionic currents of myocardial repolarization. Inhibition of IKr and Ito leads to prolonged cardiomyocyte repolarization, which in turn causes prolonged action potential duration (APD), leading to early after-depolarizations (EAD) and triggering activity, ultimately inducing arrhythmia 10, 55. IL-6 and IL-1 also activate L-Ca^2+^ channels (ICaL) on the myocardial membrane, resulting in increased Ca^2+^ inflow and prolonged action potential plateau, which in turn leads to prolonged APD 59, 61. TNF-α can inhibit Ito and Ikr, and slow delayed rectifying potassium channel (IKs), resulting in prolonged APD or QT interval and promoting arrhythmia 10, 55. Secondly, inflammatory cytokines, especially IL-1, IL-6 and TNF-α, can also lead to intracellular calmodulin dysfunction such as ryanodine receptor 2 (RYR2) and sarcoplasmic reticulum Ca^2+^-ATPase (SERCA), resulting in increased spontaneous calcium leakage, intracellular calcium overload and abnormal intracellular calcium processing during diastole, inducing delayed after-depolarization (DAD) and arrhythmia 56. Inflammatory cytokines, particularly TNF-α, have been shown to induce abnormal expression and/or distribution of gap junction proteins connexin (CX) 40 and 43 in cardiomyocytes. This disruption of the gap junction proteins can lead to dysfunction in cell-to-cell communication, affecting the conduction of cardiac electrical activity 10, 55. As a result, it can cause delayed conduction and increased heterogeneity in conduction, thereby promoting ectopic excitation or re-entry, which are common mechanisms underlying arrhythmias 55.

Cytokine storms can also lead to coagulation dysfunction and fibrinolytic system imbalance, causing pulmonary embolism, right heart overload and right atrium enlargement, induced endothelial injury, rupture of atherosclerotic plaque, formation of microthrombus, myocardial ischemia, myocardial microinfarction or infarction; meanwhile, inflammatory cytokines can activate myofibroblasts to induce the synthesis of extracellular matrix, thus leading to myocardial fibrosis; all of these lead to cardiac remodeling and increased susceptibility to arrhythmia (**Figure 3C**) 54. In addition, cytokines can also affect the parasympathetic and sympathetic nerves system, destroying electrical conduction in the heart, and further leading to the development of arrhythmia 10, 56. Inflammatory cytokines such as IL-1, IL-6 and IFN-γ can also cause metabolic disorders of liver cytochrome P450, affect the clearance rate of the body, prolong the action time of some antiviral drugs that prolong the QT interval, resulting in a marked extension of the QT interval in patients with COVID-19 and an increase in susceptibility to TdP (**Figure 3C**) 10, 62.

Overall, dysregulation of immune response and cytokine storm contribute to arrhythmia by causing electrical and structural remodeling of the heart. Cardiac electrical remodeling is caused by dysfunction of myocardial ion channels, impaired gap connections between cardiomyocytes, abnormal calcium processing, autonomic dysfunction and drug metabolic rate disorder 10, 55, 56. Suppressing immune response disorders and cytokine storm are crucial for treating multiple organ dysfunction and reducing the incidence of arrhythmia in patients with COVID-19, which may play a role of 'killing two birds with one stone' 10.

### Myocarditis

There are numerous studies showing that SARS-CoV-2 can cause myocarditis 63, 64, however, the specific mechanism remains unclear. It is hypothesized that myocarditis may arise from both direct viral destruction of cardiac cells and the disruption of the inflammatory immune system of the host 40, 50, 63, 65, 66. The incidence of COVID-19-related myocarditis is unknown because definitive diagnosis of myocarditis relies on myocardial biopsy and cardiac MRI, which are rarely used in clinical diagnosis 67. The Centers for Disease Control and Prevention reported that the prevalence of myocarditis in patients with COVID-19 is less than 0.2% of all cases 68, however, about 7% of COVID-19-related deaths are due to myocarditis 69.

Arrhythmia is one of the clinical manifestations of viral myocarditis, and the risk of arrhythmia in COVID-19-related myocarditis is high. It has been reported in the literature that 78.7% of patients with myocarditis had arrhythmia 21, 63, 70. Fulminant myocarditis can induce various malignant arrhythmias and threaten life 71. COVID-19-related myocarditis can induce arrhythmia through various mechanisms. Firstly, disruption of the plasma membrane may result in myocardial electrical instability due to direct damage to cardiomyocytes. Secondly, microvascular dysfunction can cause damage to endothelial cells, leading to myocardial ischemia. Additionally, dysfunction of intercellular gap junctions and abnormal expression of connexin due to myocardial cell injury can occur. It is noteworthy that inflammation can contribute to the formation of myocardial fibrosis or scar tissue, thereby restructuring the myocardium. Lastly, cytokines can disrupt calcium homeostasis and ion channel function, resulting in prolonged action potential repolarization, abnormal conduction and the development of triggering activity or re-entry formation 21, 66. It is noteworthy that these mechanisms are suggested possibilities, and further research is required to fully understand the complex relationship between COVID-19-related myocarditis and arrhythmia development.

### Myocardial ischemia and hypoxia

SARS-CoV-2 infects lung tissue by binding to ACE2 receptors in type II alveolar epithelial cells, resulting in lung injury, hypoxemia and acute respiratory failure 12. It has been reported that 32% of patients with COVID-19 develop hypoxemia, and 76% require oxygen support, such as mask oxygen, mechanical ventilation or even extracorporeal oxygenation using membrane lungs 21, 40. The patient's low oxygen saturation leads to myocardial hypoxia and ischemia 12. At the same time, the body's inflammatory response and immune system are activated due to SARS-CoV-2 infection, resulting in vascular endothelial cell damage and internal dermatitis. This leads to the abnormal activation of platelets and clotting factors, with the body entering a hypercoagulable state and promoting thrombosis. Thrombosis can potentially lead to myocardial ischemia or even myocardial infarction 12, 27, 44, 72.

Myocardial ischemia and hypoxia can indeed impair the function of various cardiac ion channels, transporters and ion pumps, including sodium, potassium and calcium ion channels, sodium-calcium exchangers, and sodium-potassium ATPase. The dysfunction of these critical components can lead to disturbances in ion concentrations inside and outside the cardiomyocytes, as well as changes in transmembrane potential 73. These alterations disrupt the normal autorhythmicity, excitability and conductivity of cardiomyocytes, ultimately resulting in abnormal electrical activity within the heart. This disruption of normal electrical activity markedly increases the risk of developing arrhythmias 73, 74. Hypoxemia can cause vasoconstriction of pulmonary arteries, resulting in elevated pulmonary artery pressure. The increased pulmonary artery pressure further burdens the right heart, leading to right heart dysfunction 75. Long-term myocardial ischemia and hypoxia can lead to the formation of scar tissue and fibrosis within the myocardial tissue. These can cause remodeling of cardiac structure, leading to cardiac conduction dysfunction, promoting triggering activity and re-entry 13, 21.

### Electrolyte abnormalities and intravascular volume imbalance

The arrhythmia effects of electrolyte abnormalities have been studied extensively 40. Electrolyte disorders can disrupt the dynamics of ionic currents in the heart, leading to changes in the concentration of ions inside and outside the cardiomyocytes. These changes can have a notable impact on the electrophysiological function of cardiomyocytes, potentially resulting in arrhythmias of varying severity, some of which may be life-threatening 76. Imbalance in ACE2 expression and overactivation of the RAAS system caused by SARS-CoV-2 invasion, along with gastrointestinal dysfunction, secondary renal tubular dysfunction due to viral invasion and excessive release of inflammatory factors, all of which can potentially result in electrolyte abnormalities 77. A case study involving 416 patients with COVID-19 revealed that 7.2% of them experienced electrolyte disturbances 21. In a retrospective analysis, it was found that 27% of hospitalized patients with COVID-19 suffered from acute kidney injury, which may cause electrolyte disturbance 40. Electrolyte abnormalities, including hypokalemia, hyponatremia and hypocalcemia, are frequently observed in patients with COVID-19 21. In patients with severe COVID-19, intravascular volume imbalance is a common clinical manifestation 21. This imbalance can be attributed to conditions such as acute respiratory distress syndrome or sepsis-induced heart failure 21. Atrial fibrillation is the most commonly observed type of arrhythmia in severe patients with COVID-19 and its occurrence may be associated with intravascular volume imbalance. Excessive volume load can lead to atrial stretching and atrial structural remodeling 76. Intravascular volume imbalance can also cause changes in plasma osmotic pressure, which in turn leads to overactivation of sympathetic nerves and increases susceptibility to arrhythmia 21.

### Drug toxicity and interactions and new coronary vaccine

The pharmacological treatments for COVID-19 include oral small molecule drugs such as azulfidine, paxlovid, lopinavir/ritonavir, molnupiravir, fabiprevir, baricitinib, as well as chloroquine phosphate, monoclonal antibodies, glucocorticoids, IL-6 inhibitors and antibacterial drugs 21, 77. Lopinavir/ritonavir, chloroquine phosphate, hydroxychloroquine, azithromycin (AZM) can inhibit human ether-à-go-go related gene (hERG) channels, lead to ventricular repolarization or QT interval prolongation, and cause ventricular arrhythmias and even ventricular fibrillation 78. Chloroquine phosphate, an antimalarial drug, is also used in the treatment of COVID-19 79. However, it is noteworthy that chloroquine has the potential to prolong the QT interval, resulting in life-threatening arrhythmias such as ventricular tachycardia or ventricular fibrillation 16, 79. Similarly, hydroxychloroquine, another medication used for COVID-19 treatment, can induce alternating repolarization and initiate polymorphic ventricular tachycardia 45. Chloroquine and hydroxychloroquine can inhibit the cytochrome P450 metabolic pathway, cause the decrease of drug clearance, strengthen the action time of drugs that prolong the QT interval and increase the risk of arrhythmia 10, 21. In a retrospective study, it was observed that patients who received a combination of hydroxychloroquine and AZM had higher rates of the corrected QT(QTc) prolongation (33%) compared with those patients who were treated with hydroxychloroquine alone (5%) 56. AZM belongs to the macrolide class of antimicrobials and is known to be associated with an increased risk of arrhythmia and cardiac death when used alone or in combination with lopinavir/ritonavir and chloroquine 80. Long-term use of AZM has been shown to increase the late cardiac sodium current and elevate the concentration of sodium ions in cardiomyocytes. This results in the extrusion of sodium ions out of the cell through sodium-calcium exchanger on the cell membrane, while simultaneously allowing an influx of calcium ions into the cell. This sustained increase in intracellular calcium can lead to calcium overload, ultimately causing the prolongation of the QT interval 45, 81. COVID-19 vaccines, especially mRNA-based ones, have been associated with vaccine-induced myocarditis, leading to myocardial damage and potential arrhythmias 21. A global study on COVID-19 vaccination revealed an increased risk of myocarditis, pericarditis and arrhythmias in patients who contracted SARS-CoV-2 after receiving the vaccine 82.

### Autonomic dysfunction

After SARS-CoV-2 infection, patients may experience various stress-related conditions such as mental stress, anxiety, fatigue, pain, quarantine and sleep disorders 70. These conditions, combined with the effects of medications, can lead to dysfunction in the autonomic nervous system 70. This dysfunction, along with the activation of the sympatho-adrenal medulla axis and the hypothalamic-pituitary-adrenal cortex axis, results in the release of large amounts of catecholamines; consequently, vasoconstriction and cardiotoxicity can occur, potentially leading to myocardial damage or malignant arrhythmia 21, 70. Additionally, the excessive activation of inflammatory factors and the marked increase of IL-6 can stimulate the central hypothalamus, leading to the overactivation of the cardiac sympathetic system and increasing the risk of developing arrhythmias 83.

## Conclusion

At present, the situation of the COVID-19 epidemic is generally stable, with a notable decrease in the number of people infected with SARS-CoV-2, and severe or critically ill patients. However, the severity of the disease should not be underestimated and patients with underlying diseases are still at high risk of contracting the virus. In the future, it is possible that individuals will continue to coexist with SARS-CoV-2, and may experience multiple infections with the virus. Continued vigilance and adherence to public health guidelines are required to prevent COVID-19 and to mitigate virus-induced arrhythmias. At the same time, controlling the underlying disease status, early detection and treatment of myocardial damage and other organ dysfunction, avoiding hypoxia and using drugs to prolong the QT interval, avoiding systemic inflammation, and seeking psychological treatment to relieve tension and anxiety may be beneficial for alleviating COVID-19-related arrhythmias. Exploring the mechanisms behind COVID-19-related arrhythmias is crucial for clinicians to enhance prevention efforts, monitor arrhythmias more effectively, optimize treatment strategies and improve the overall prognosis and survival rates of patients with COVID-19. Conducting in-depth studies on the pathophysiological mechanisms of COVID-19-associated arrhythmias, such as exploring the direct or indirect effects of ion channel or transporter function, cellular oxidation and metabolic balance, may provide valuable insights for identifying potential targets and diagnostic markers for the treatment of COVID-19-related arrhythmias. Finally, it is anticipated that the present review will be useful in raising clinicians' awareness of arrhythmia in patients with COVID-19, and in promoting related research and clinical practice. Future in-depth studies are required on the relationship between COVID-19 and arrhythmias to better protect and manage the heart health in patients with COVID-19.

## Abbreviations

SARS-CoV-2: severe acute respiratory syndrome coronavirus-2
ACE2: angiotensin-converting enzyme 2
COVID-19: coronavirus disease-2019
ARDS: acute respiratory distress syndrome
RBD: receptor binding domain
TMPRSS2: trans membrane protease serine 2
RAAS: renin-angiotensin-aldosterone system
AT1: angiotensin type 1
ACEIs: angiotensin converting enzyme inhibitors
ARBs: angiotensin receptor blockers
IL: interleukin
IFN-γ: interferon -γ
TNF-α: tumor necrosis factor-α
TdP: torsades de pointes
IKr: rapid activation of delayed rectified
Ito: transient output potassium current
IKs: slow delayed rectifying potassium channel
ICaL: L-Ca^2+^ channels
NK: natural killer cell
INa: Na^+^-current
APD: action potential duration
EAD: early after-depolarization
DAD: delayed after-depolarization
CX: connexin
hERG: human ether-à-go-go related gene
RYR2: ryanodine receptor 2
SERCA: sarcoplasmic reticulum Ca^2+^-ATPase
SR: sarcoplasmic reticulum
AZM: azithromycin
QTc: corrected QT

## References

[1] Sawalha K, Abozenah M, Kadado AJ (2021). Systematic Review of COVID-19 Related Myocarditis: Insights on Management and Outcome. Cardiovasc Revasc Med.

[2] Ziegler CGK, Miao VN, Owings AH (2021). Impaired local intrinsic immunity to SARS-CoV-2 infection in severe COVID-19. Cell.

[3] Hu B, Guo H, Zhou P (2021). Characteristics of SARS-CoV-2 and COVID-19. Nat Rev Microbiol.

[4] Huang C, Wang Y, Li X (2020). Clinical features of patients infected with 2019 novel coronavirus in Wuhan, China. Lancet.

[5] Cai YT, Mason KA (2022). Why they willingly complied: Ordinary people, the big environment, and the control of COVID-19 in China. Soc Sci Med.

[6] Qu P, Evans JP, Faraone JN (2023). Enhanced neutralization resistance of SARS-CoV-2 Omicron subvariants BQ.1, BQ.1.1, BA.4.6, BF.7, and BA.2.75.2. Cell Host Microbe.

[7] Lyke KE, Atmar RL, Islas CD (2022). Rapid decline in vaccine-boosted neutralizing antibodies against SARS-CoV-2 Omicron variant. Cell Rep Med.

[8] De Gaetano A, Bajardi P, Gozzi N (2023). Behavioral Changes Associated With COVID-19 Vaccination: Cross-National Online Survey. J Med Internet Res.

[9] Saniasiaya J, Narayanan P (2022). Parosmia post COVID-19: an unpleasant manifestation of long COVID syndrome. Postgrad Med J.

[10] Lazzerini PE, Boutjdir M, Capecchi PL (2020). COVID-19, Arrhythmic Risk, and Inflammation: Mind the Gap!. Circulation.

[11] Nishiga M, Wang DW, Han Y (2020). COVID-19 and cardiovascular disease: from basic mechanisms to clinical perspectives. Nat Rev Cardiol.

[12] Gupta A, Madhavan MV, Sehgal K (2020). Extrapulmonary manifestations of COVID-19. Nat Med.

[13] Babapoor-Farrokhran S, Gill D, Walker J (2020). Myocardial injury and COVID-19: Possible mechanisms. Life Sci.

[14] Boulos PK, Freeman SV, Henry TD (2023). Interaction of COVID-19 With Common Cardiovascular Disorders. Circ Res.

[15] Ali S, Khanal R, Najam M (2024). Short-Term Outcomes of Cardiac Arrhythmias Among COVID-19 Patients: A Propensity Matched National Study. Curr Probl Cardiol.

[16] Guzik TJ, Mohiddin SA, Dimarco A (2020). COVID-19 and the cardiovascular system: implications for risk assessment, diagnosis, and treatment options. Cardiovasc Res.

[17] Zheng YY, Ma YT, Zhang JY (2020). COVID-19 and the cardiovascular system. Nat Rev Cardiol.

[18] Driggin E, Madhavan MV, Bikdeli B (2020). Cardiovascular Considerations for Patients, Health Care Workers, and Health Systems During the COVID-19 Pandemic. J Am Coll Cardiol.

[19] Chen N, Zhou M, Dong X (2020). Epidemiological and clinical characteristics of 99 cases of 2019 novel coronavirus pneumonia in Wuhan, China: a descriptive study. Lancet.

[20] Wang Y, Wang Z, Tse G (2020). Cardiac arrhythmias in patients with COVID-19. J Arrhythm.

[21] Zhan Y, Yue H, Liang W (2022). Effects of COVID-19 on Arrhythmia. J Cardiovasc Dev Dis.

[22] Yang H, Rao Z (2021). Structural biology of SARS-CoV-2 and implications for therapeutic development. Nat Rev Microbiol.

[23] Ning Q, Wu D, Wang X (2022). The mechanism underlying extrapulmonary complications of the coronavirus disease 2019 and its therapeutic implication. Signal Transduct Target Ther.

[24] V'Kovski P, Kratzel A, Steiner S (2021). Coronavirus biology and replication: implications for SARS-CoV-2. Nat Rev Microbiol.

[25] Lan J, Ge J, Yu J (2020). Structure of the SARS-CoV-2 spike receptor-binding domain bound to the ACE2 receptor. Nature.

[26] Walls AC, Park YJ, Tortorici MA (2020). Structure, Function, and Antigenicity of the SARS-CoV-2 Spike Glycoprotein. Cell.

[27] Shang J, Ye G, Shi K (2020). Structural basis of receptor recognition by SARS-CoV-2. Nature.

[28] Piccoli L, Park YJ, Tortorici MA (2020). Mapping Neutralizing and Immunodominant Sites on the SARS-CoV-2 Spike Receptor-Binding Domain by Structure-Guided High-Resolution Serology. Cell.

[29] Jackson CB, Farzan M, Chen B (2022). Mechanisms of SARS-CoV-2 entry into cells. Nat Rev Mol Cell Biol.

[30] Trigueiro-Louro J, Correia V, Figueiredo-Nunes I (2020). Unlocking COVID therapeutic targets: A structure-based rationale against SARS-CoV-2, SARS-CoV and MERS-CoV Spike. Comput Struct Biotechnol J.

[31] Hamming I, Timens W, Bulthuis ML (2004). Tissue distribution of ACE2 protein, the functional receptor for SARS coronavirus. A first step in understanding SARS pathogenesis. J Pathol.

[32] Yan H, Jiao H, Liu Q (2021). ACE2 receptor usage reveals variation in susceptibility to SARS-CoV and SARS-CoV-2 infection among bat species. Nat Ecol Evol.

[33] Viana Martins CP, Xavier CSF, Cobrado L (2022). Disinfection methods against SARS-CoV-2: a systematic review. J Hosp Infect.

[34] Higuchi Y, Suzuki T, Arimori T (2021). Engineered ACE2 receptor therapy overcomes mutational escape of SARS-CoV-2. Nat Commun.

[35] Yang J, Petitjean SJL, Koehler M (2020). Molecular interaction and inhibition of SARS-CoV-2 binding to the ACE2 receptor. Nat Commun.

[36] Donoghue M, Hsieh F, Baronas E (2000). A novel angiotensin-converting enzyme-related carboxypeptidase (ACE2) converts angiotensin I to angiotensin 1-9. Circ Res.

[37] Fleg JL, Aronow WS, Frishman WH (2011). Cardiovascular drug therapy in the elderly: benefits and challenges. Nat Rev Cardiol.

[38] Tomasoni D, Adamo M, Lombardi CM (2019). Highlights in heart failure. ESC Heart Fail.

[39] Welsh TJ, Gladman JR, Gordon AL (2014). The treatment of hypertension in people with dementia: a systematic review of observational studies. BMC Geriatr.

[40] Dherange P, Lang J, Qian P (2020). Arrhythmias and COVID-19: A Review. JACC Clin Electrophysiol.

[41] Saha SA, Russo AM, Chung MK (2022). COVID-19 and Cardiac Arrhythmias: a Contemporary Review. Curr Treat Options Cardiovasc Med.

[42] Baggen J, Jacquemyn M, Persoons L (2023). TMEM106B is a receptor mediating ACE2-independent SARS-CoV-2 cell entry. Cell.

[43] Triggle CR, Bansal D, Ding H (2021). A Comprehensive Review of Viral Characteristics, Transmission, Pathophysiology, Immune Response, and Management of SARS-CoV-2 and COVID-19 as a Basis for Controlling the Pandemic. Front Immunol.

[44] Donniacuo M, De Angelis A, Rafaniello C (2023). COVID-19 and atrial fibrillation: Intercepting lines. Front Cardiovasc Med.

[45] Manolis AS, Manolis AA, Manolis TA (2020). COVID-19 infection and cardiac arrhythmias. Trends Cardiovasc Med.

[46] Xu SC, Wu W, Zhang SY (2022). Manifestations and Mechanism of SARS-CoV2 Mediated Cardiac Injury. Int J Biol Sci.

[47] Bhatla A, Mayer MM, Adusumalli S (2020). COVID-19 and cardiac arrhythmias. Heart Rhythm.

[48] Yang L, Liu S, Liu J (2020). COVID-19: immunopathogenesis and Immunotherapeutics. Signal Transduct Target Ther.

[49] Chung MK, Zidar DA, Bristow MR (2021). COVID-19 and Cardiovascular Disease: From Bench to Bedside. Circ Res.

[50] Bojkova D, Wagner JUG, Shumliakivska M (2020). SARS-CoV-2 infects and induces cytotoxic effects in human cardiomyocytes. Cardiovasc Res.

[51] Han Y, Zhu J, Yang L (2022). SARS-CoV-2 Infection Induces Ferroptosis of Sinoatrial Node Pacemaker Cells. Circ Res.

[52] Wong LR, Perlman S (2022). Immune dysregulation and immunopathology induced by SARS-CoV-2 and related coronaviruses - are we our own worst enemy?. Nat Rev Immunol.

[53] Du P, Geng J, Wang F (2021). Role of IL-6 inhibitor in treatment of COVID-19-related cytokine release syndrome. Int J Med Sci.

[54] Lazzerini PE, Laghi-Pasini F, Boutjdir M (2022). Inflammatory cytokines and cardiac arrhythmias: the lesson from COVID-19. Nat Rev Immunol.

[55] Lazzerini PE, Laghi-Pasini F, Boutjdir M (2019). Cardioimmunology of arrhythmias: the role of autoimmune and inflammatory cardiac channelopathies. Nat Rev Immunol.

[56] Lazzerini PE, Capecchi PL, Laghi-Pasini F (2017). Systemic inflammation and arrhythmic risk: lessons from rheumatoid arthritis. Eur Heart J.

[57] Wang J, Wang H, Zhang Y (2004). Impairment of HERG K(+) channel function by tumor necrosis factor-alpha: role of reactive oxygen species as a mediator. J Biol Chem.

[58] Sawaya SE, Rajawat YS, Rami TG (2007). Downregulation of connexin40 and increased prevalence of atrial arrhythmias in transgenic mice with cardiac-restricted overexpression of tumor necrosis factor. Am J Physiol Heart Circ Physiol.

[59] Aromolaran AS, Srivastava U, Alí A (2018). Interleukin-6 inhibition of hERG underlies risk for acquired long QT in cardiac and systemic inflammation. PLoS One.

[60] Monnerat G, Alarcón ML, Vasconcellos LR (2016). Macrophage-dependent IL-1beta production induces cardiac arrhythmias in diabetic mice. Nat Commun.

[61] Hagiwara Y, Miyoshi S, Fukuda K (2007). SHP2-mediated signaling cascade through gp130 is essential for LIF-dependent I CaL, [Ca2+]i transient, and APD increase in cardiomyocytes. J Mol Cell Cardiol.

[62] El-Ghiaty MA, Shoieb SM, El-Kadi AOS (2020). Cytochrome P450-mediated drug interactions in COVID-19 patients: Current findings and possible mechanisms. Med Hypotheses.

[63] Fairweather D, Beetler DJ, Di Florio DN (2023). COVID-19, Myocarditis and Pericarditis. Circ Res.

[64] Patone M, Mei XW, Handunnetthi L (2022). Risks of myocarditis, pericarditis, and cardiac arrhythmias associated with COVID-19 vaccination or SARS-CoV-2 infection. Nat Med.

[65] Siripanthong B, Nazarian S, Muser D (2020). Recognizing COVID-19-related myocarditis: The possible pathophysiology and proposed guideline for diagnosis and management. Heart Rhythm.

[66] Peretto G, Sala S, Rizzo S (2019). Arrhythmias in myocarditis: State of the art. Heart Rhythm.

[67] Castiello T, Georgiopoulos G, Finocchiaro G (2022). COVID-19 and myocarditis: a systematic review and overview of current challenges. Heart Fail Rev.

[68] Boehmer TK, Kompaniyets L, Lavery AM (2021). Association Between COVID-19 and Myocarditis Using Hospital-Based Administrative Data - United States, March 2020-January 2021. MMWR Morb Mortal Wkly Rep.

[69] Ruan Q, Yang K, Wang W (2020). Clinical predictors of mortality due to COVID-19 based on an analysis of data of 150 patients from Wuhan, China. Intensive Care Med.

[70] DePace NL, Colombo J (2022). Long-COVID Syndrome and the Cardiovascular System: A Review of Neurocardiologic Effects on Multiple Systems. Curr Cardiol Rep.

[71] Nair A, Deswal A (2022). COVID-19-Associated Fulminant Myocarditis: Pathophysiology-Related Phenotypic Variance. J Am Coll Cardiol.

[72] Wu CI, Postema PG, Arbelo E (2020). SARS-CoV-2, COVID-19, and inherited arrhythmia syndromes. Heart Rhythm.

[73] Yang KC, Kyle JW, Makielski JC (2015). Mechanisms of sudden cardiac death: oxidants and metabolism. Circ Res.

[74] El-Sherif N, Turitto G (2011). Electrolyte disorders and arrhythmogenesis. Cardiol J.

[75] Naeije R, Richter MJ, Rubin LJ (2022). The physiological basis of pulmonary arterial hypertension. Eur Respir J.

[76] Shaver CM, Chen W, Janz DR (2015). Atrial Fibrillation Is an Independent Predictor of Mortality in Critically Ill Patients. Crit Care Med.

[77] Wang RS, Loscalzo J (2023). Repurposing Drugs for the Treatment of COVID-19 and Its Cardiovascular Manifestations. Circ Res.

[78] Zequn Z, Yujia W, Dingding Q (2021). Off-label use of chloroquine, hydroxychloroquine, azithromycin and lopinavir/ritonavir in COVID-19 risks prolonging the QT interval by targeting the hERG channel. Eur J Pharmacol.

[79] Husayn SS, Brown JD, Presley CL (2022). Hydroxychloroquine Alternatives for Chronic Disease: Response to a Growing Shortage Amid the Global COVID-19 Pandemic. J Pharm Pract.

[80] Varney JA, Dong VS, Tsao T (2022). COVID-19 and arrhythmia: An overview. J Cardiol.

[81] Noble D, Noble PJ (2006). Late sodium current in the pathophysiology of cardiovascular disease: consequences of sodium-calcium overload. Heart.

[82] Abutaleb MH, Makeen HA, Meraya AM (2023). Risks of Cardiac Arrhythmia Associated with COVID-19 Vaccination: A Systematic Review and Meta-Analysis. Vaccines (Basel).

[83] Manolis AA, Manolis TA, Apostolopoulos EJ (2021). The role of the autonomic nervous system in cardiac arrhythmias: The neuro-cardiac axis, more foe than friend?. Trends Cardiovasc Med.

